# Evidence of Territoriality and Species Interactions from Spatial Point-Pattern Analyses of Subarctic-Nesting Geese

**DOI:** 10.1371/journal.pone.0081029

**Published:** 2013-12-02

**Authors:** Matthew E. Reiter, David E. Andersen

**Affiliations:** 1 Minnesota Cooperative Fish and Wildlife Research Unit, Department of Fisheries, Wildlife, and Conservation Biology, University of Minnesota, Saint Paul, Minnesota, United States of America; 2 U.S. Geological Survey, Minnesota Cooperative Fish and Wildlife Research Unit, Saint Paul, Minnesota, United States of America; University of Missouri-Columbia, United States of America

## Abstract

Quantifying spatial patterns of bird nests and nest fate provides insights into processes influencing a species’ distribution. At Cape Churchill, Manitoba, Canada, recent declines in breeding Eastern Prairie Population Canada geese (*Branta canadensis interior*) has coincided with increasing populations of nesting lesser snow geese (*Chen caerulescens caerulescens*) and Ross’s geese (*Chen rossii*). We conducted a spatial analysis of point patterns using Canada goose nest locations and nest fate, and lesser snow goose nest locations at two study areas in northern Manitoba with different densities and temporal durations of sympatric nesting Canada and lesser snow geese. Specifically, we assessed (1) whether Canada geese exhibited territoriality and at what scale and nest density; and (2) whether spatial patterns of Canada goose nest fate were associated with the density of nesting lesser snow geese as predicted by the protective-association hypothesis. Between 2001 and 2007, our data suggest that Canada geese were territorial at the scale of nearest neighbors, but were aggregated when considering overall density of conspecifics at slightly broader spatial scales. The spatial distribution of nest fates indicated that lesser snow goose nest proximity and density likely influence Canada goose nest fate. Our analyses of spatial point patterns suggested that continued changes in the distribution and abundance of breeding lesser snow geese on the Hudson Bay Lowlands may have impacts on the reproductive performance of Canada geese, and subsequently the spatial distribution of Canada goose nests.

## Introduction

The spatial distribution of nesting birds is likely influenced by multiple factors that vary in their importance among spatial scales and exhibit potentially complex interactions [1], [2]. At a local spatial scale (e.g., nest level [high resolution, small extent]), habitat attributes and access to food resources, inter- and intra-specific interactions, and predator pressure likely influence spatial patterns of nesting birds [3]-5, whereas at a regional spatial scale (i.e., low resolution, large extent), climate and geomorphology are also likely to be important [2]. The magnitude of the effect of these factors is directly related to their influence on the vital rates of nesting birds; primarily the survival of the nest or the survival of the nesting bird [6], [7]. Changes in climate or geomorphology may affect range-wide reproductive performance leading to shifts in nesting distribution across an entire species’ range. However, these interactions likely occur over a long temporal extent, and subsequently are difficult to predict or manage [2]. Changes in the spatial distribution of nests over a shorter temporal extent are likely driven by local spatial-scale interactions. Describing the spatial distributions of nests, quantifying spatial variation in vital rates (e.g., nest success), and identifying short-term changes in nesting patterns at the local spatial scale can provide insight into processes influencing a species’ distribution [6], [8].

Long-term monitoring (>30 years) of breeding Eastern Prairie Population (EPP) Canada geese (*Branta canadensis interior*) at Cape Churchill, Manitoba, Canada has documented a localized decline in nest density [9], [10]; however, across the EPP Canada goose nesting range, the breeding population has remained relatively stable or increased [10]. This suggests there has been a shift in the spatial distribution of nesting Canada geese in this region. Concurrently, nesting lesser snow geese (*Chen caerulescens caerulescens*) and Ross’s geese (*Chen rossii*) have expanded their spatial distribution along western Hudson Bay [11], moving into areas that were traditionally used solely by nesting EPP Canada geese [10]. At the spatial scale of the EPP Canada goose range, Canada geese and lesser snow geese utilize similar coastal tundra nest habitat and occur in high densities across this landscape [10]-[12]. However, at a more local spatial scale, lesser snow geese are colony nesters (spatial aggregation) and Canada geese typically exhibit dispersed (spatial inhibition; i.e., maximize distance between nests) nesting ecology [9], [13]-[15] although they have also been considered “semi-colonial” [15].

The impact of increasing densities of nesting lesser snow geese on nesting Canada geese is not well known. Previous studies have identified two patterns. On Akimski Island, Nunavut, Canada geese experienced reduced nest survival when nesting among lesser snow geese [16]. In a different study, cackling geese (*Branta huchinsii*) showed evidence, in part, supporting a protective association with higher nest success for cackling goose nests within aggregations of nesting Ross’s geese [17]. However, cackling goose nest survival was reduced when there were very high densities of Ross’s goose nests within 30 m. A recent range-wide evaluation of nesting Canada geese and lesser snow geese along western Hudson Bay and Cape Churchill [18] identified landscape-scale patterns in the distribution of breeding EPP Canada geese consistent with the findings in the cackling goose-Ross’s goose study [17].

Spatial statistics for point patterns provide a rigorous format for quantifying spatial distributions, testing simple hypotheses, and examining potential interactions among nesting birds [19]-[21]. Analyses of spatial point patterns primarily utilize inter-point distances over a selected region [20]. These distances are compared to those simulated using a Poisson (random) process. Nearest-neighbor analysis incorporates the distance from each point to its nearest-neighbor point [22] and provides information about local-scale or first-order spatial patterns [23]. Ripley’s *K*-function quantifies spatial patterns across multiple spatial scales and incorporates information about the expected count of all points at different distances from each point of interest to assess second-order patterns [23]. First-order and second-order bivariate analyses can also test for independence in the spatial point patterns of two or more types (e.g., two species, successful versus failed nests) [19], [20]. The use of both first- and second-order analyses provides insight as to the mechanism of spatial interactions. Significant associations in first-order nearest-neighbor interactions suggest potential local interactions among geese from individual nests, which may be indicative of territoriality. Significant associations in second-order analyses provide an assessment of potential interactions associated with total abundance of nests. Evaluation of protective associations among nesting geese [17], which are likely influenced by overall abundance of birds rather than the proximity of nearest-neighbors, may be better examined with second-order analyses.

We employed spatial point-pattern analyses to quantify patterns of Canada goose nests, Canada goose nest fate, and lesser snow goose nests at two study areas in northern Manitoba, Canada with different temporal durations (5 – 6 years and 10 – 15 years) of sympatric nesting Canada and lesser snow geese. First, using first-order analysis, we tested the hypothesis that Canada geese exhibit dispersed nesting ecology and territoriality. Second, by examining two study areas with differences in the temporal duration of sympatric nesting Canada geese and lesser snow geese and second-order point-pattern analyses, we tested the protective-association hypothesis as a mechanism influencing the observed spatial distribution of Canada goose nests relative to lesser snow goose nests.

## Materials and Methods

### Study area

**Figure 1:**
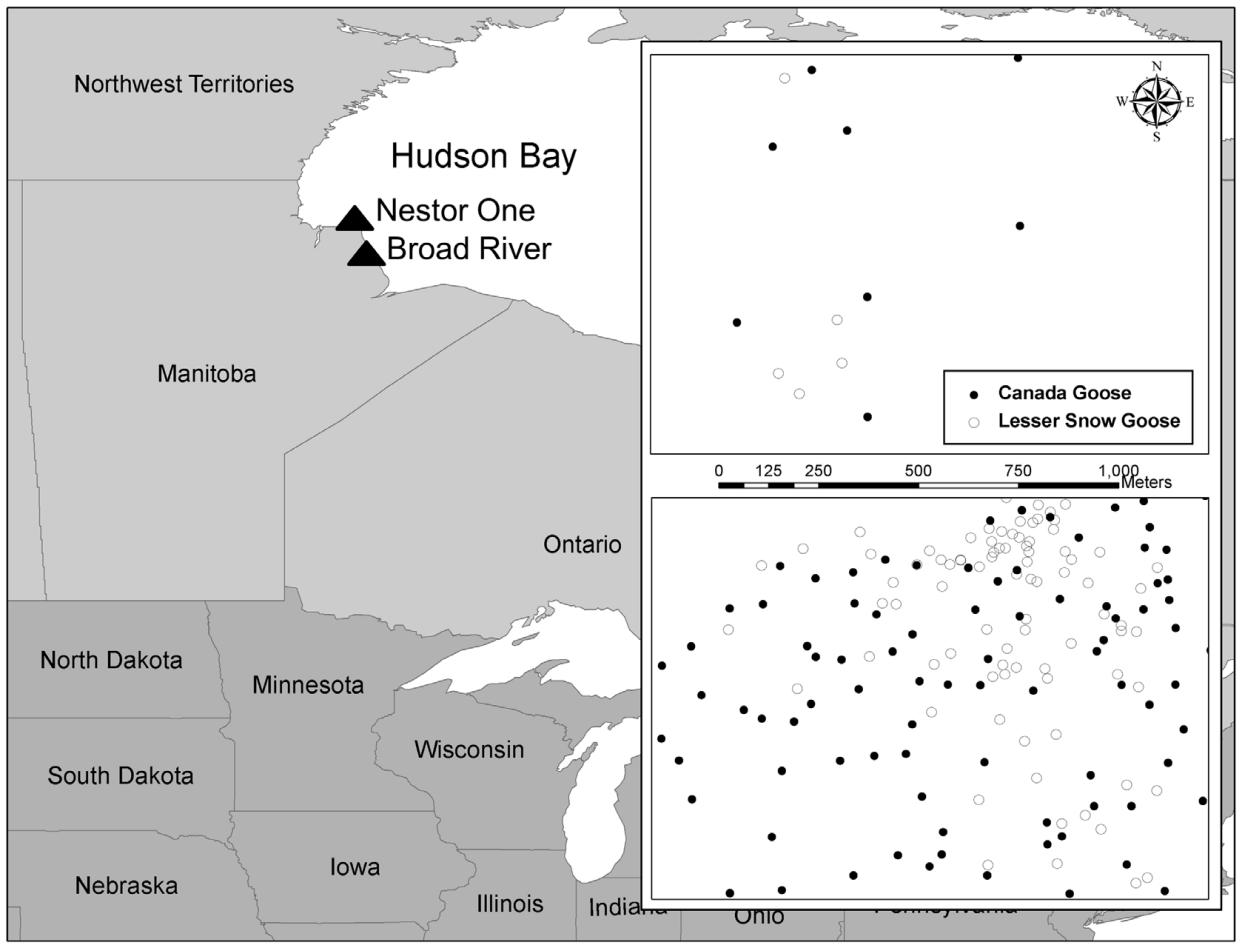
Location of Nestor One and Broad River study areas in northern Manitoba, Canada. Inset panels represent a 1.5-km^2^ area of Nestor One (top) and Broad River (bottom) and associated distribution of Canada goose nests and lesser snow goose nests in an average year, 2005.

The EPP Canada goose breeding range includes ∼101,500 km^2^ of northern Manitoba, Canada [24]. The highest density of breeding EPP Canada geese is found along a strip of coastal tundra bordering western Hudson Bay [10], [12]. The Nestor One study area (∼48 km^2^; 58° 34’ N, 93° 11’ W) was south of Cape Churchill and approximately 60 km east-southeast of the town of Churchill, Manitoba (Fig. 1). Nesting lesser snow geese (>5 nests) have been present at Nestor One beginning in 2001, however breeding lesser snow geese from a colony 20-km west have used Nestor One for brood-rearing for more than 30 years. The Broad River study area (∼10 km^2^; 58° 07’ N, 92° 51’ W) was located ∼60 km south of Nestor One along the Hudson Bay coast (Fig. 1). While relatively few lesser snow goose nests were documented during ground surveys in the mid-1990s [9], annual aerial breeding-ground surveys documented a >200% increase in the number of lesser snow goose nests near the mouth of the Broad River since 2000. Overall, Broad River, on average, had much higher densities of both Canada goose nests and lesser snow goose nests than Nestor One (Fig. 1). Nestor One and Broad River were located inside of Wapusk National Park (11,475 km^2^) within the broader ecosystem of the Hudson Bay Lowlands.

### Nest distribution and fate

We conducted systematic ground surveys for nesting Canada geese and lesser snow geese at Nestor One (2000–2007) and Broad River (2005–2006) following standardized protocols [10]. Nest observers carried global positioning system (GPS) units to record the Universal Transverse Mercator (UTM) coordinates of Canada goose and lesser snow goose nests. At Nestor One and Broad River, we estimated the hatch date for all nests using egg flotation and egg candling [25]–[27] and returned to nests at or subsequent to the estimated hatch date to determine nest fate. We classified Canada goose nest fate as successful (≥1 egg hatched) or failed [10]. Due to low rates of nest abandonment in EPP Canada geese and the strong influence of the abundance of predators on nest survival [9], we considered failure rate of Canada goose nests in our study to represent a depredation rate.

### Analyses of point patterns

Irregular study area boundaries and highly heterogeneous distribution of habitats can confound analysis of spatial point patterns [20], [23]. We imported Canada goose and lesser snow goose nest data into R v.2.5.1 (© 2007 The R Foundation for Statistical Computing; use of trade names does not imply endorsement by either the U.S. Geological Survey or the University of Minnesota) and used the SPATSTAT package for point-pattern analysis [23]. This software permitted increased flexibility and reality in the analysis of point patterns, allowing for irregular study area borders and incorporating areas within a study region where no events can occur (e.g., a lake). Both Nestor One and Broad River are irregularly shaped and at Nestor One areas not used by nesting geese (e.g., water and large beach ridges) divide the study area. We used ArcMAP 9.0 (© 1998 – 2004 ESRI) and a vegetation classification layer to delineate water and beach ridges at Nestor One and Broad River [28]. We considered water bodies and inland beach ridges >1 ha in size as patches of non-habitat. We coded non-habitat patches into the image we created in R representing Nestor One. The Broad River was highly homogenous with only small bodies of water. We did not include any non-habitat patches in our analyses for the Broad River.

For Canada goose and lesser snow goose nests at each study area in each year, we conducted a first-order nearest-neighbor analysis and a second-order Ripley’s-*K* analysis. The calculation of the empirical nearest-neighbor *G*-estimate was:
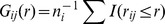



Where, 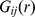 was the probability a nest of type *i* has a nearest neighbor of type *j* at distance, *r; n_i_* was the total number of nests of type *i*; *I* was an indicator variable assigned a value of 1 if 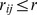 and 0 otherwise; and 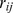 was the distance from nest of type *i* to its nearest neighbor of type *j* [29], [30].

The calculation of the Ripley’s-*K* estimate, *K_ij_*(*r*), was:
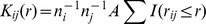



Where, *n_j_* was the total events of type *j*, and *A* was the area (m^2^) of the study region. All remaining variables were the same as defined for 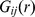. We employed these equations (*G*-estimate and *K*-estimate) for both univariate (type *i*  =  type *j*) and bivariate (type *i* ≠ type *j*) analyses below.

We compared the observed test statistic, *K_ij_*(*r*) or *G_ij_*(*r*), against the distribution of *K_ij_*(*r*) or *G_ij_*(*r*) from 199 permutations of point patterns based on a Poisson-process model with the same density as the observed nests [31]. The 5^th^ and 195^th^ ranked values of the simulated statistic at each distance we evaluated formed the 95% critical envelope (95% CE). We graphed the 95% CE to test for significant deviations from complete spatial randomness in each of our analyses. At each distance, observed *K_ij_*(*r*) or *G_ij_*(*r*) below the 95% CE indicated significant deviations from complete spatial randomness towards regularity or spatial inhibition at the *α*  =  0.05 significance level. Observed *K_ij_*(*r*) or *G_ij_*(*r*) above the 95% CE indicated significant aggregation.

We predicted that if Canada geese exhibited dispersed nesting and territoriality then first-order Canada goose nest distribution should reveal spatial inhibition. However, a mixed strategy of first-order inhibition and second-order aggregation might suggest local-scale territoriality coupled with selection of habitat or other factors influencing broader-scale patterns.

For analyses of the distribution of successful and failed Canada goose nests, we employed a random labeling simulation [20]. This technique assumed the spatial distribution of all Canada goose nests, whether they eventually failed or succeeded, was generated by the same underlying process. We tested whether given this underlying distribution of nests the marks (successful or failed) were distributed randomly in space. We constructed the 95% CE by simulating 199 permutations of the point pattern when we assigned marks (i.e. nest fates) randomly.

Lastly, we utilized multi-type nearest-neighbor and Ripley’s *K* analysis, which assumed nests of each type (i.e., species), came from their own underlying spatial process, to evaluate whether Canada goose nest fate was correlated with its spatial positioning relative to lesser snow goose nests. We evaluated successful and failed Canada goose nests separately, relative to all lesser snow goose nests.

We considered second-order aggregation of successful Canada goose nests with lesser snow goose nests and second-order inhibition between failed Canada goose nests and lesser snow goose nests as evidence in support of the protective-association hypothesis. This hypothesis would be further supported if short-term spatial patterns of Canada goose nest fate and lesser snow goose nests at Nestor One suggested aggregation between successful Canada goose nests and lesser snow goose nests, coupled with data from longer-term sympatric nesting at Broad River that identified a positive association between Canada goose nests and lesser snow goose nests. Alternatively, first-order aggregation of failed Canada goose nests with lesser snow goose nests but second-order aggregation between successful Canada goose nests and lesser snow goose nests would be consistent with Baldwin et al. [17]. This would suggest that at a local scale (e.g., nearest neighbor) Canada geese do not benefit from a nearby nesting lesser snow goose. However, at a broader spatial scale, when incorporating information about the overall density of nesting lesser snow geese, Canada goose nests are more likely to be successful if aggregated with lesser snow geese than if located away from lesser snow goose aggregations.

### Assumption

Analyses of spatial point patterns typically assume all events (e.g., nests) were detected within a selected region (probability of detection  =  1). However, the theoretical calculation of Ripley’s *K*-estimate (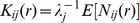) is the ratio of the expected count of points in an area defined by a circle of radius, *r* (E[*N_ij_*(*r*)]) under complete spatial randomness, and the mean nest density (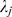). Therefore, if a random sample of the pattern is removed, the resulting *K*-estimate is proportional to the *K*-estimate of the entire spatial pattern. Essentially, “random thinning” [20] multiplies both values in the ratio by the probability (*p*) that one event is kept. Walter and Rusch [32] reported the probability of discovering an EPP Canada goose nest was 0.72, and importantly, that there was no spatial variation in this probability at Nestor One. For our analyses, we assumed 0.72 was equivalent to *p* and thus the observed spatial sample of nests represented a random and spatially unbiased subset of the full point pattern. A similar argument applies in nearest-neighbor analysis in which the theoretical distribution function of *G_ij_* [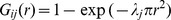], under complete spatial randomness, is defined by 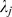. Therefore, removing a random selection of proportion *p* of the nests does not change the shape of the distribution of *G_ij_*.

To facilitate interpretation of summary statistics, we converted 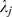 from nests per m^2^ (used in point-pattern analysis calculations) to nests per km^2^. Because the detection probabilities of a Canada goose nest and lesser snow goose nest are likely different, we did not directly compare the relative nest density (RND) between species.

This study was conducted under strict adherence to protocol 0802A27490 approved by the University of Minnesota Animal Care and Use Committee and did not include any protected or endangered species. All field work occurred within Wapusk National Park, Manitoba, Canada under Parks Canada Agency Research and Collection Permits WAP-2005-503 and WAP 2005-518. Data from this research can be made available through a direct request to the corresponding author.

## Results

### Nest summary

At Nestor One, there were >5 lesser snow goose nests each year during 2001–2003 and 2005–2007. We used these six years in analyses of point patterns at Nestor One, and data from 2005 and 2006 at the Broad River. The average density of Canada goose nests was substantially lower (5.2 nests per km^2^, SE  =  0.30, *n*  =  6) at Nestor One (Fig. 1) than at Broad River (42.5 nests per km^2^, SE  =  0.45, *n*  =  2; Table 1 and Fig. 1). Lesser snow goose nest density was also lower at Nestor One (1.3 nests per km^2^; SE  =  0.45, *n*  =  6) than the Broad River (22.4 nest per km^2^, SE  =  6.35, *n*  =  2; Table 1 and Fig. 1). Apparent nest success (successful nests/total number of nests) averaged 0.49 for Canada geese at Nestor One between 2000 and 2007 (although an average of 0.73 in 2005 and 2006) and 0.71 at Broad River in 2005 and 2006. Overall, lesser snow goose nests experienced low nest success at Nestor One (0.23) between 2002 and 2007. In 2005 and 2006 nest success for lesser snow geese (0.50) was still lower than at Broad River (0.63) for the same years (Table 1). The year with the highest success of lesser snow goose nests at Nestor One, 2003, corresponded with their highest nest total.

**Table 1:** Number of nests (*n*), nest fates (S  =  successful; F  =  failed), and relative nest density (RND; nests per km^2^) for Eastern Prairie Population Canada geese and lesser snow geese nests at two study areas near Cape Churchill, in northern Manitoba, Canada between 2001 and 2007.

		Canada geese				Lesser snow geese			
AREA	YEAR	*n*	RND	S	F	*n*	RND	S	F
Nestor One	2001	160	5.24	132	28	42	1.38	-	-
	2002	118	3.87	39	79	6	0.20	0	6
	2003	152	4.98	92	60	91	2.98	46	45
	2005	173	5.67	128	45	68	2.23	18	50
	2006	177	5.80	127	50	18	0.59	1	17
	2007	173	5.67	93	80	13	0.43	4	9
Broad River	2005	403	41.90	291	104	154	16.00	87	60
	2006	413	43.00	284	129	276	28.70	182	94

### Intra-specific spatial patterns of nests

**Figure 2:**
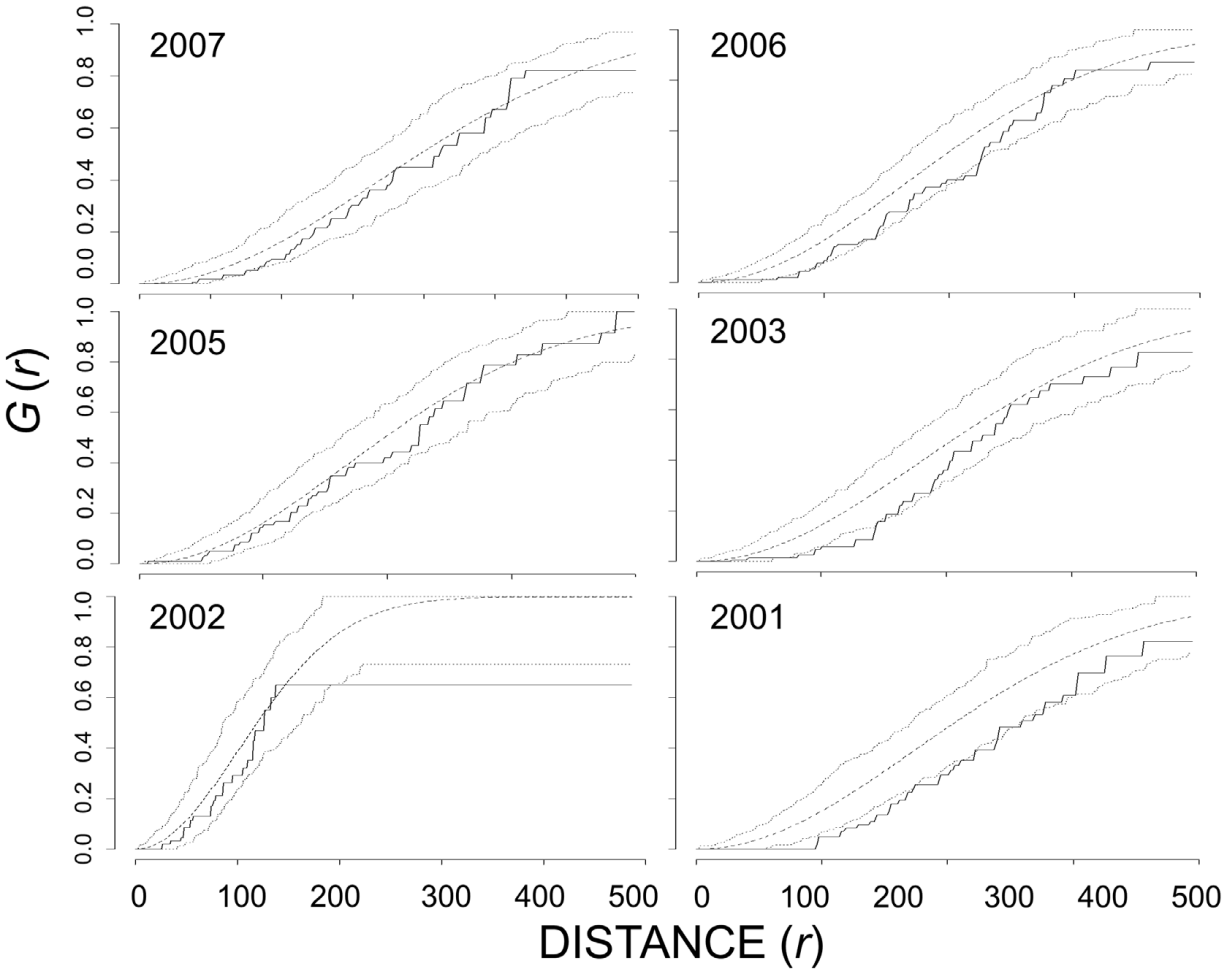
Nearest-neighbor analysis, G(*r*), of all Canada goose nests at Nestor One 2001 – 2003, 2005 – 2007. The dashed line represents the theoretical distribution under complete spatial randomness, the dotted lines are the upper and lower 95% critical envelope (CE), and the solid black line is the observed distribution of 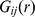. Observed distribution above the 95% CE indicated aggregation whereas those below the 95% CE indicated inhibition. Distances are in meters.

**Figure 3:**
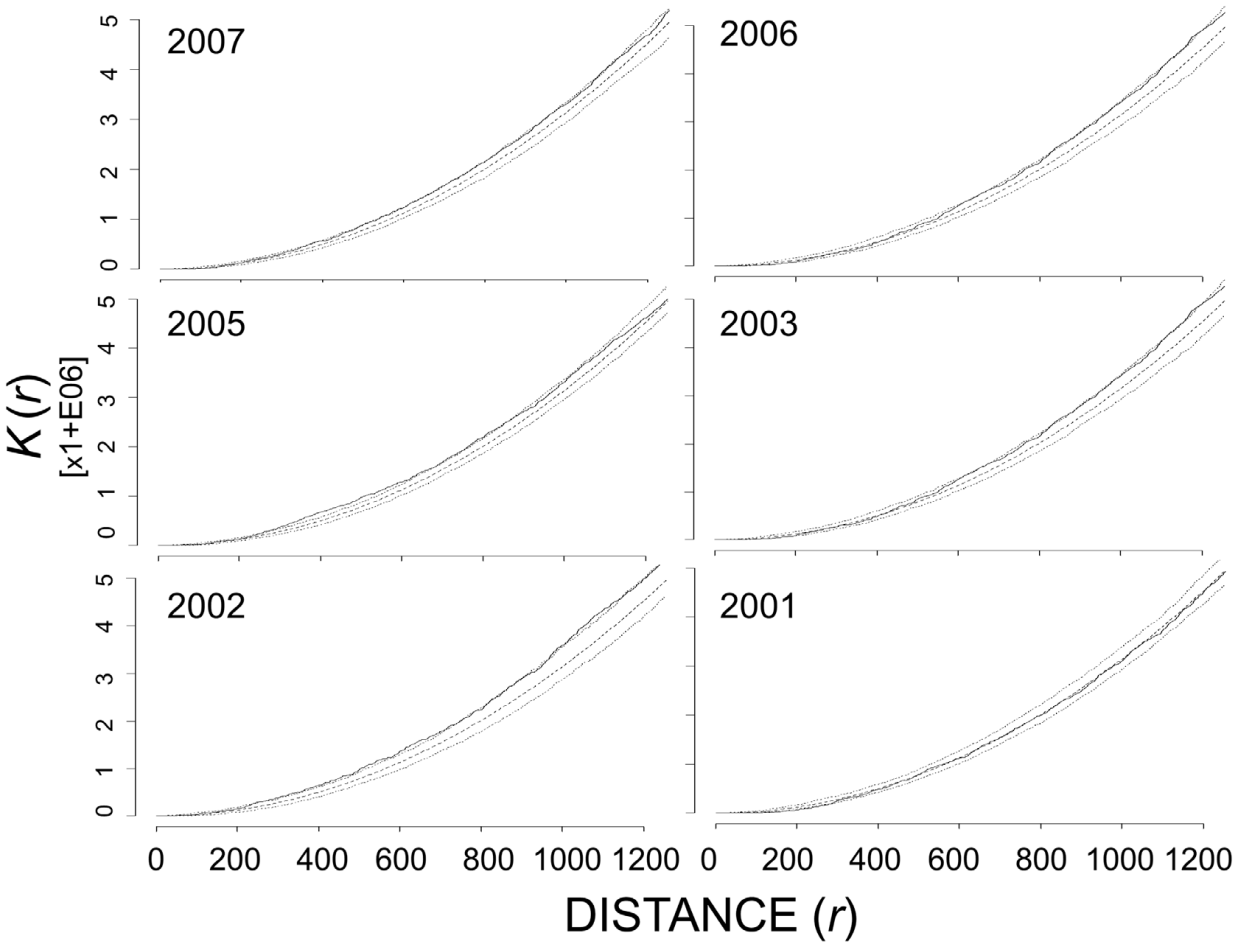
Ripley’s *K*-estimate of all Canada goose nests at Nestor One, 2001 – 2003, 2005 – 2007. The dashed line represents the theoretical distribution under complete spatial randomness, the dotted lines are the upper and lower 95% critical envelope (CE), and the solid black line is the observed distribution of *K_ij_*(*r*). Observed distribution above the 95% CE indicated aggregation whereas those below the 95% CE indicated inhibition. Distances are in meters.

**Figure 4:**
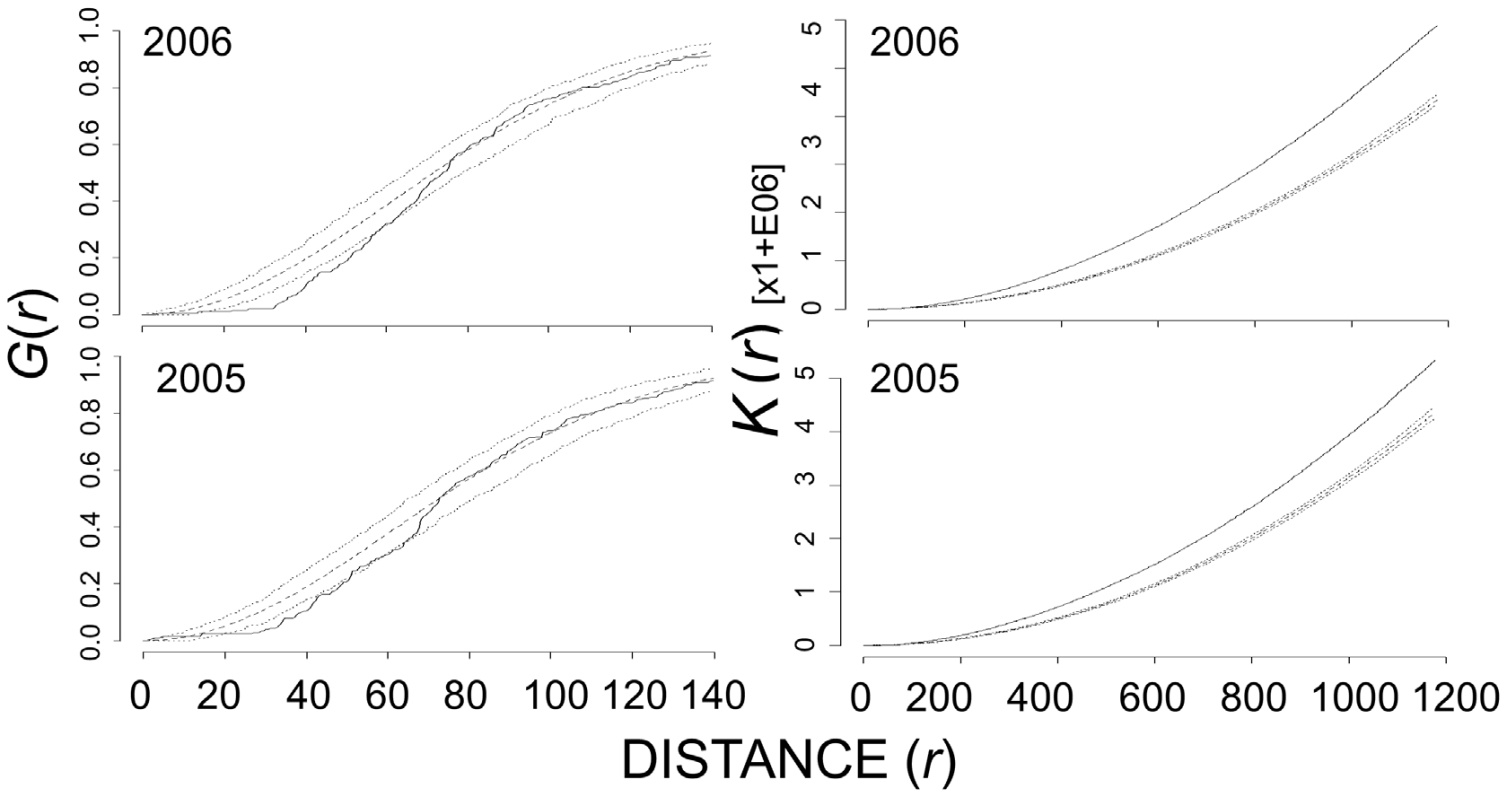
Nearest-neighbor, G(*r*), (left column), and Ripley’s *K*-estimate (right column) of all Canada goose nests at Broad River, 2005 – 2006. The dashed line represents the theoretical distribution under complete spatial randomness, the dotted lines are the upper and lower 95% critical envelope (CE), and the solid black line is the observed distribution of 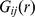 or *K_ij_*(*r*). Observed distribution above the 95% CE indicated aggregation whereas those below the 95% CE indicated inhibition. Distances are in meters.

Overall, Canada goose nests exhibited significant deviations towards inhibition in first-order analyses and significant aggregation in second-order analyses (Table 2). At Nestor One, in three (2001, 2003, 2006) of six years, Canada goose nests exhibited significant deviations from complete spatial randomness towards inhibition based on the distribution of nearest neighbors between 75 and 200 m (Fig. 2). However, in second-order analyses, the *K*_ij_(*r*) for all Canada goose nests fell above the 95% CE, tending toward aggregation, across many distances in five of six years (Table 2 and Fig. 3). At Broad River in 2005 and 2006, Canada goose nests exhibited significant inhibition at ∼20 – 60 m in first-order analysis and significant aggregation at all distances evaluated in second-order analysis (Fig. 4).

**Table 2:** Percentage of first-order (nearest neighbor) and second-order (*K*-estimate) spatial point pattern analyses (eight possible year-site combinations) of Canada geese (CAGO) and lesser snow geese (LSGO) nests at two study areas near Cape Churchill, in northern Manitoba, Canada that were statistically significant (P < 0.05) and suggested either aggregation (+) or inhibition (−).

	CAGO	CAGOsuccess^1^	CAGOfail^2^	CAGOsuccess	CAGOfail	LSGO
First-order Analysis						
CAGO	63% (−)					
CAGO		25% (−)				
CAGO			25% (+)			
LSGO				38% (+)		
LSGO					88% (+)12% (−)	
LSGO						75% (+)
Second-order Analysis						
CAGO	88% (+)					
CAGO		25% (+)				
CAGO			88% (+)			
LSGO				50% (+)12% (−)		
LSGO					75% (+)12% (−)	
LSGO						88% (+)

The distribution of columns was compared to the distribution of rows in all analyses.

1 success  =  distribution of successful nests.

2 fail  =  distribution of failed nests.

**Figure 5:**
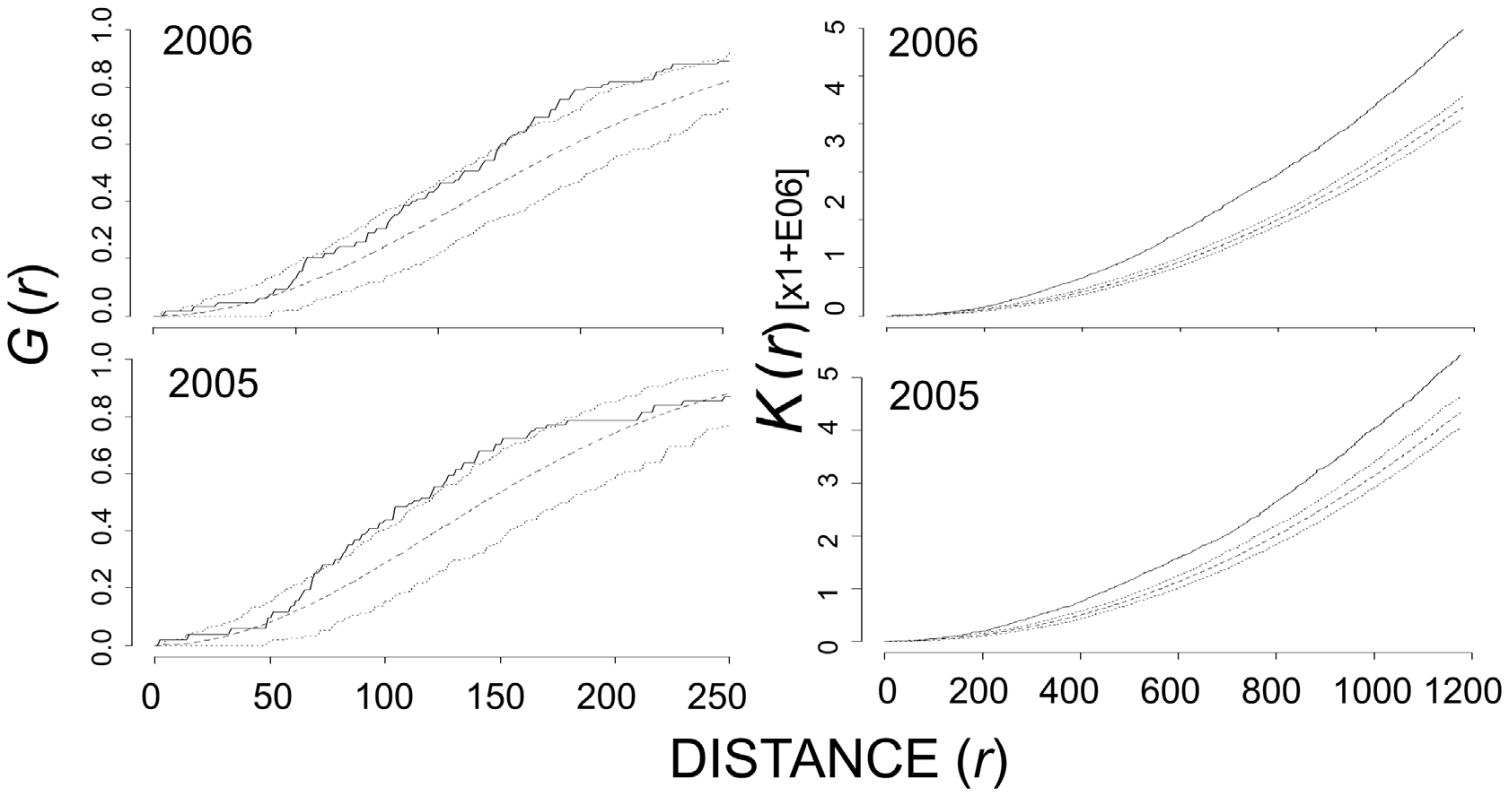
Nearest-neighbor, G(*r*), (left column) and Ripley’s *K*-estimate (right column) of failed Canada goose nests at Broad River, 2005 – 2006. The dashed line represents the theoretical distribution under complete spatial randomness, the dotted lines are the upper and lower 95% critical envelope (CE), and the solid black line is the observed distribution of 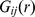
or *K_ij_*(*r*). Observed distribution above the 95% CE indicated aggregation whereas those below the 95% CE indicated inhibition. Distances are in meters.

Generally, we observed aggregation in the distribution of successful and failed Canada goose nests relative to a random pattern of nest fate (Table 2). Given the underlying spatial distribution of Canada goose nests at Nestor One, the spatial pattern of successful Canada goose nests did not deviate from random labeling in any year we evaluated in either first- or second-order analyses. At Broad River in 2005 and 2006, successful Canada goose nests exhibited spatial inhibition at local (∼20 – 50 m) scales, and significant aggregation at all distances in second-order analyses. Although failed Canada goose nests at Nestor One exhibited few trends in first-order analysis, second-order analysis indicated significant deviations from random labeling towards aggregation for at least some distances in five of six years. Within the second order analysis at Nestor One, we observed significant aggregation of failed nests at scales of 400 – 600 m in 2002 and 2006 and at broader scales of 800 – 1,200 m in 2003, 2005, and 2007. At Broad River in 2005 and 2006, failed Canada goose nests were aggregated at ∼100 – 200 m in first-order analysis and at all distances in second-order analyses in both years (Fig. 5).

Overall, lesser snow goose nests were aggregated (Table 2) at both Nestor One and Broad River. Lesser snow goose nests at Nestor One tended significantly towards aggregation in four of six years evaluated based on first-order analyses, in five of six years based on the second-order *K*-estimate, and across nearly all distances. At the Broad River, lesser snow goose nests were significantly aggregated across nearly all distances in all analyses.

### Inter-specific spatial patterns of nests

Successful Canada goose nests, overall, tended towards aggregation with lesser snow goose nests but only significantly in 50% of comparisons (Table 2). At Nestor One, we observed few significant deviations (one of six years) from complete spatial randomness in the relationship between successful Canada goose nests and lesser snow goose nests in first-order analysis. Second-order deviations from complete spatial randomness occurred in 2003, 2005, and 2006. In 2003 and 2006, there was significant aggregation between successful Canada goose nests and lesser snow goose nests. In 2005, successful Canada goose nests exhibited significant inhibition at 1,000 – 1,200 m with lesser snow goose nests. At Broad River, successful Canada goose nests were significantly aggregated with lesser snow goose nests across nearly all distances in both first- and second-order analyses in 2005 and 2006.

**Figure 6:**
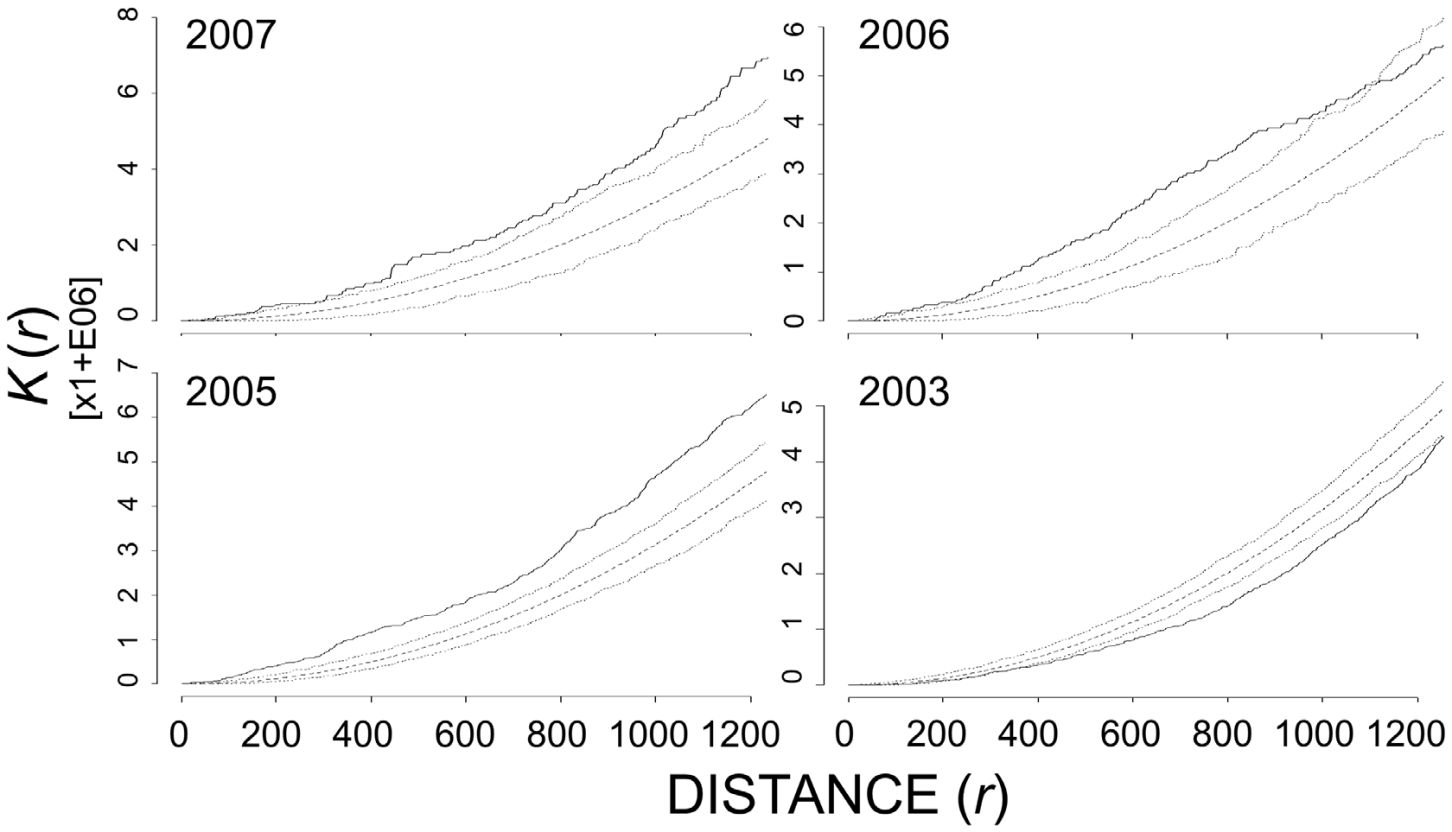
Multi-type Ripley’s *K*-estimate of failed Canada goose nests relative to all lesser snow goose nests at Nestor One, 2003, 2005 – 2007. The dashed line represents the theoretical distribution under complete spatial randomness, the dotted lines are the upper and lower 95% critical envelope (CE), and the solid black line is the observed distribution of *K_ij_*(*r*). Observed distribution above the 95% CE indicated aggregation whereas those below the 95% CE indicated inhibition. Distances are in meters.

Overall, failed Canada goose nests exhibited strong aggregation with lesser snow geese nests (Table 2). At Nestor One, failed Canada goose nests and lesser snow goose nests exhibited significant, albeit variable, interactions at some distances in all six years based on first-order analysis. There was significant aggregation between failed Canada goose nests and lesser snow goose nests, 2005 – 2007; however, in 2003, there was significant inhibition between failed Canada goose nests and lesser snow goose nests. In second-order analyses for Nestor One, the observed pattern between failed Canada goose nests and lesser snow goose nests deviated substantially from complete spatial randomness across many distances in four of six years (Fig. 6). From 2005 to 2007 there was significant aggregation; however, in 2003 the relationship was exactly opposite with significant inhibition between failed Canada goose nests and lesser snow goose nests. At Broad River, failed Canada goose nests were aggregated with lesser snow goose nests at all distances in both first- and second-order analyses in 2005 and 2006.

## Discussion

The relative spatial positioning and changes in the distribution of two breeding bird species can offer evidence concerning intra- and inter-specific interactions [19], [33]. Our quantification of the spatial distribution of Canada goose nests, Canada goose nest fates, and lesser snow goose nests near Cape Churchill, Manitoba using spatial point-pattern analyses identified several strong non-random patterns in nest distribution and nest fate. We also partially confirmed the protective-association hypothesis as a mechanism influencing interactions between nesting Canada geese and lesser snow geese over the last 10 – 15 years. The probability of nest failure (depredation) in Canada geese nesting sympatrically with lesser snow geese was conditional on lesser snow goose nest density. We observed aggregation between failed Canada goose nests and lesser snow goose nests at Nestor One in first- and second-order analysis, and inhibition between successful Canada goose nests and lesser snow goose nests in second-order analysis when density of lesser snow geese was low (2005 – 2007). When density of snow goose nests was high (2003) successful Canada goose nests were aggregated with lesser snow goose nests. Similarly, at Broad River, where lesser snow goose nest density was much higher than at Nestor One and temporal duration of sympatric nesting has been longer, successful Canada goose nests tended to be aggregated strongly with lesser snow goose nests in both first- and second-order analysis. Combined, these results suggest the effect of lesser snow goose nests on Canada goose nest fate may change depending on overall density of lesser snow goose nests. This pattern, in part, supported the protective-association hypothesis as it applies to interactions between nesting Canada geese and lesser snow geese in northern Manitoba.

Although spatial patterns of northern-nesting Canada geese have been described as “highly territorial” [15], few studies have addressed the spatial scale at which the territorial pattern was realized and whether this pattern was dependent upon the density of nests. Our first-order spatial analyses provide some of the first quantitative evidence that tundra-nesting Canada geese are territorial. However, overall our results suggest that the scale of territoriality and spatial patterns of Canada goose nests may differ depending on the spatial scale of the observations. The spatial distribution of EPP Canada goose nests varied from dispersed to aggregated across spatial scales between 2001 and 2007; exhibiting significant nearest-neighbor (∼20 – 50 m) intra-specific inhibition, while significantly aggregating with con-specifics at broader spatial scales (>1,000 m). Local-scale inhibition across the range of nest densities observed at both Nestor One and Broad River supports the hypothesis that Canada geese exhibit local-scale territoriality and identifies the spatial extent of territories in densely nesting Canada geese in a tundra landscape. The cause(s) of broader scale aggregation were likely related to the distribution of available habitat, access to resources, the distribution of predators, or associations with conspecifics and other species. Ultimately, there may be multiple factors, which vary among spatial scales and in their effect on nest distributions, driving the spatial patterns of Canada goose nests.

The protective-association hypothesis predicts that if Canada goose nests experienced increased nest success through reduced nest depredation (nearly 100% of Canada goose nests that fail are depredated in this ecosystem) when associated with aggregations of lesser snow goose nests, over time Canada goose nests would tend toward further aggregation with lesser snow goose nests. This prediction was partially confirmed by the strong aggregations of Canada goose and lesser snow goose nests at Broad River where Canada geese and lesser snow geese have been nesting sympatrically for ≥10 years, particularly in second-order analyses. The nest densities of both species have increased during this period [9], [10] with now some of the highest densities of Canada goose nests recorded in arctic and subarctic regions occurring at Broad River. In addition, at Nestor One, failed Canada goose nests were strongly aggregated with lesser snow goose nests in years with very low lesser snow goose nest density but occurred farther from lesser snow geese in a year with high density of lesser snow goose nests. This difference in the relationship between Canada goose nest fate and lesser snow goose nests at Nestor One was accompanied by declining numbers of nesting snow geese. In 2003, there were 91 snow goose nests at Nestor One, and from 2005–2007 this number declined from 69 to 13 (Table 1).

Large numbers of nesting birds are often necessary to successfully establish a new colony when group defense and predator protection play an important role in nesting ecology [34]. In Alaska, nests of black brant (*Branta bernicla*) experienced lower rates of depredation in colonies with >100 nests [35]. At the La Pèrouse Bay colony (>20,000 nesting pairs) near Churchill, Manitoba researchers documented high nest success and low depredation rates in densely nesting lesser snow geese [13]. In our study, lesser snow geese experienced substantially lower apparent nest success when nesting at low densities at Nestor One compared to higher densities at Broad River (Table 1). It may be that Canada geese and lesser snow geese only begin to benefit from spatial associations when aggregations of lesser snow goose nests reach a level that provides the benefits of group defense from predators. Aggregations of nests below this threshold density may attract predators and increase the likelihood of nest failure for both species.

In a recent study, the probability of nest survival in cackling geese was positively associated with a nest being located within a Ross’s goose colony but negatively associated with the number of Ross’s goose nests within 30 m [17]. Our first- and second-order analysis of successful Canada goose nests relative to lesser snow goose nests at Broad River identified aggregation at both scales. The difference in results between these studies may be the result of considerably lower lesser snow goose nest densities in our analysis, than that of Ross’s goose nests in Baldwin et al. [17], thus limiting the number of lesser snow goose nests within short distances of Canada goose nests. If the density of nesting lesser snow geese continues to increase at Broad River, to nest density levels of Ross’s geese and lesser snow geese observed at other locations [13], [17], the relationship between Canada goose nest fate and nearest-neighboring lesser snow goose nest may shift from positive and aggregation to negative and inhibition. A range-wide analysis of spatial patterns of nesting Canada geese and lesser snow geese in this region identified that, similar to this study, there was correlation between Canada goose and lesser snow goose nest densities [18]. However, once lesser snow goose nest density was above 40 nests per km^2^ there was a negative association between Canada goose nest density and lesser snow goose nest density. In our study, the highest observed density of lesser snow geese was 26 nests per km^2^ at Broad River in 2006. The range-wide study [18] would have predicted strong aggregation of Canada goose nests and lesser snow goose nests at the densities observed in our study.

Lesser snow geese nested in aggregations at both Nestor One and Broad River, and significant aggregation of lesser snow goose nests occurred across all densities of nests we evaluated. Furthermore, we identified different trends in lesser snow goose nest abundance between Nestor One and Broad River. At Nestor One, lesser snow goose nest density increased dramatically in the early 2000s but steadily declined in recent years. Factors limiting the expansion of the lesser snow goose nest aggregations at Nestor One are not known; however, the importance of adequate coastal salt marsh vegetation for lesser snow geese has been well documented [36]. Recent declines in lesser snow goose nest density at La Pèrouse Bay, ∼20 km west of Nestor One, coincided with the loss of salt marsh vegetation [11], [13]. At Nestor One, the majority of salt marsh habitat has been altered by brood-rearing lesser snow geese that travel from La Pèrouse Bay, suggesting that this area may be unable to support high densities of nesting lesser snow geese. Conversely, the density of lesser snow goose nests at Broad River, which was much greater than at Nestor One during our study but substantially less than observed elsewhere in the Arctic [37]–[39], has grown substantially since 1995 when few lesser snow goose nests were located in ground-based searches [9], [10]. Currently, the status of coastal salt marshes near Broad River is not well known, limiting comparisons of possible mechanisms causing the differential lesser snow goose nest density trends observed between our study areas.

Increases in the number of lesser snow geese along western Hudson Bay have had “catastrophic” impacts on coastal tundra vegetation [11]. However, less attention has been dedicated to their potential impacts on other vertebrate species. Analyses of spatial point patterns provided a rigorous framework to test hypotheses regarding spatial patterns of nesting Canada geese and lesser snow geese, and processes influencing these patterns. Our results suggested the probability of Canada goose nest failure was influenced by the size and distribution of lesser snow goose nest aggregations. Spatial variation in probability of nest failure was related to the overall density of lesser snow goose nests and thus may also vary with time. Whether this variation will ultimately influence the evolution of nesting strategies in Canada geese (i.e., dispersed vs. aggregated) was unclear, despite evidence from Broad River that Canada geese and lesser snow geese may nest successfully in the same dense aggregations. Overall, density-dependent trends in the relative spatial distribution of these two tundra-nesting species and Canada goose nest fate provide critical insights into the long-term implication of lesser snow goose population expansions on Canada geese.
